# An Efficient Methodology Combining K-Means Machine Learning and Electrochemical Modelling for the Determination of Ionic Diffusivity and Kinetic Properties in Battery Electrodes

**DOI:** 10.3390/ma16145146

**Published:** 2023-07-21

**Authors:** Odile Capron, Luis D. Couto

**Affiliations:** 1 Vlaamse Instelling voor Technologisch Onderzoek (VITO) NV, Boeretang 200, 2400 Mol, Belgium; luis.coutomendonca@vito.be; 2 EnergyVille, Thor Park 8310, 3600 Genk, Belgium

**Keywords:** Galvanostatic Intermittent Titration Technique (GITT), unsupervised machine learning, k-means clustering, electrochemical model, pseudo-two-dimensional, sodium ion, NVPF, diffusion coefficient, reaction rate constant

## Abstract

This paper presents an innovative and efficient methodology for the determination of the solid-state diffusion coefficient in electrode materials with phase transitions for which the assumption of applying the well-known formula from the work of Weppner et al. is not satisfied. This methodology includes a k-means machine learning screening of Galvanostatic Intermittent Titration Technique (GITT) steps, whose outcomes feed a physics-informed algorithm, the latter involving a pseudo-two-dimensional (P2D) electrochemical model for carrying out the numerical simulations. This methodology enables determining, for all of the 47 steps of the GITT characterization, the dependency of the Na^+^ diffusion coefficient as well as the reaction rate constant during the sodiation of an NVPF electrode to vary between 9 × $10^{-18}$ and 6.8 × $10^{-16}$ m^2^·s^−1^ and between 2.7 × $10^{-14}$ and 1.5 × $10^{-12}$ m^2.5^·mol^−0.5^·s^−1^, respectively. This methodology, also validated in this paper, is (a) innovative since it presents for the first time the successful application of unsupervised machine learning via k-means clustering for the categorization of GITT steps according to their characteristics in terms of voltage; (b) efficient given the considerable reduction in the number of iterations required with an average number of iterations equal to 8, and given the fact the entire experimental duration of each step should not be simulated anymore and hence can be simply restricted to the part with current and a small part of the rest period; (c) generically applicable since the methodology and its physics-informed algorithm only rely on “if” and “else” statements, i.e., no particular module/toolbox is required, which enables its replication and implementation for electrochemical models written in any programming language.

## 1. Introduction

Nowadays, there is a wide and further increasing use of lithium-ion batteries (LIBs) in mobile and stationary applications, such as electric vehicles or large-scale electric energy storage system installations. In this sense, LIBs are expected to face major challenges in meeting the actual and further growing consumer demand [1,2]. These challenges are associated with the availability limitation of LIBs in the long term and the high price of the raw materials required for their manufacturing. In view of this problem, sodium-ion batteries (SIBs) present several advantages. Among them, sodium resources have wide geographic availability and low cost and can be safely transported in their discharge state, and there are similar physicochemical properties between the two alkali Na and Li metals [3,4,5]. A SIB comprises a positive electrode made of a material commonly containing Na, a negative electrode material that does not necessarily contain Na, an electrolyte (whether liquid or solid), as well as a separator [6]. During the charge or desodiation process, sodium ions are de-inserted from the positive electrode in order to be further inserted into the negative electrode. Meanwhile, the current is flowing in the opposite direction via an external circuit. Similarly, during the discharge or sodiation of the positive electrode, sodium ions are moving from the negative electrode to go back into the positive electrode [4]. This reversible intercalation process of ions into host electrode materials is referred to as the rocking-chair working principle of battery electrochemical energy storage systems [7]. Further, the material synthesis methods of sodium compounds can easily be borrowed and adapted from lithium compounds [4]. The pouch, prismatic, or cylindrical geometrical configurations used for LIBs can be further employed for manufacturing SIBs. Hence, despite different charge carriers between SIBs (Na^+^) and LIBs (Li^+^), cell components and electrochemical ion insertion/extraction mechanisms are basically identical for both battery technologies [8]. This enabled the rapid growth of research and development activities in the field of SIB [4,5]. The accurate knowledge of ion transport properties in electrode materials is of great importance for the optimization of batteries and their performances [9,10]. Regarding the redox potential, sodium exhibits a higher-standard electrode potential than lithium (−2.71 vs. −3.02 V), involving a thermodynamic minimum limit for the negative electrode materials used in SIBs, and further leading to a lower energy density compared to LIBs [4]. In the battery energy storage market, SIBs are considered a very promising technology as well as a relevant choice and an efficient alternative to LIBs for applications in low-speed electric vehicles and large-scale stationary electric energy storage systems [11,12].

In order to develop suitable batteries for these applications, the key to battery electrochemical performance generally is the positive electrode materials [13,14,15]. However, these materials intrinsically have an insulating-like nature (poor electronic mobility) and relatively low ionic diffusion properties that can prevent them from reaching high rates and power performances [13]. In SIBs, the charge carrier Na^+^ has a larger radius than Li^+^ (1.02 Å vs. 0.76 Å) as well as a heavier atom weight (23 g·mol^−1^ vs. 6.94 g·mol^−1^) [6,16]. However, the mass of the charge carrier represents a small percentage of the overall weight of the electrode material components, such that the difference in the theoretical specific capacity of the electrodes of both SIB and LIB technologies becomes smaller [4]. Nevertheless, the differences in dimensions of the charge carrier and atom weight lead to a larger ion transport channel for SIBs, worse transport kinetics, lower phase stability with interphase formation, and low solubility in solids [17]. Tremendous efforts have been made to find appropriate electrode materials to solve these problems, leading to the development of dominant positive electrode materials for SIBs such as transition metal oxides, polyanionic compounds, Prussian blue analogs, and organic compounds [18]. Among them, fluorophosphate-based $Na_{3}V_{2}{\left(PO_{4}\right)}_{3}\;$(NVP) and $Na_{3}V_{2}{\left(PO_{4}\right)}_{2}F_{3}$(NVPF) materials are the most favorable for use as positive electrodes in SIBs due to their three-dimensional open framework, which can accelerate sodium ion transport by delivering large interstitial spaces [19]. $Na_{3}V_{2}{\left(PO_{4}\right)}_{3}\;$(NVP), with its NASICON (super-ionic conductor) structure, was revealed to be a very promising positive electrode material for SIBs given its three-dimensional sodium ion migration path, good discharge capacity, high discharge voltage plateau, high thermal stability as energy density, and excellent cycling stability [20,21]. The 3D corner-sharing structure leads to large ion diffusion channels and thus high Na^+^ diffusivity capabilities. The volume change, occurring during the intercalation and deintercalation processes of Na+ in the electrodeactive material particles, is relatively limited [21]. The NVP electrode materials exhibit a high output voltage and a stable crystal structure during both sodiation and desodiation processes [21]. During the reversible electrochemical reactions, the two sodium ions in the NVP lattice cell are intercalated and deintercalated at 3.4 V, and the theoretical discharge capacity (i.e., sodiation process) is up to 117.6 mAh·g^−1^ [22]. Techniques such as carbon material cladding modification, elemental doping, and lattice nano-sizing are further investigated to improve the electronic conductivity capabilities ($1.63\times 10^{-6}$ S·cm^−1^) of NVP electrode materials in order to target applications in large-scale energy storage systems [22].

$Na_{3}V_{2}{\left(PO_{4}\right)}_{3}\;$(NVP) and $Na_{3}V_{2}{\left(PO_{4}\right)}_{2}F_{3}$(NVPF), with its NASICON structure, are materials with large interstitial spaces allowing for the fast intercalation and deintercalation of Na^+^ ions in the electrodeactive material particles [23]. Furthermore, NVPF exhibits high energy density and outstanding stability properties. In practice, its energy density of around 475 Wh·kg^−1^ [24,25] is similar to that of LiFePO_4_ (around 580 Wh·kg^−1^) as positive electrode material in LIBs [26]. Its theoretical capacity reaches values around 128 mAh·g^−1^ [27]. In addition, the presence of strong F-V bonding leads to a high working voltage (3.7 V) vs. Na/Na^+^ [28].

Sodium ion transport inside battery (porous) electrodes is linked to the diffusion of ions in their solid phase. Therefore, sluggish Na^+^ diffusion properties can prevent NVPF material from reaching a high performance rate and long lifecycle [29]. The diffusion of ions in the electrodes can be determined and quantified through advanced electrochemical characterization techniques, namely, Cyclic Voltammetry (CV), Electrochemical Impedance Spectroscopy (EIS), Potentiostatic Intermittent Titration Technique (PITT), and the Galvanostatic Intermittent Titration Technique (GITT). In practice, diffusion coefficients calculated from CV lead to greater diffusion coefficient values compared to those determined by EIS and GITT [30]. Particularly for the latter, Weppner and Huggins [31] proposed in 1977 a convenient method to determine the diffusion coefficient of ions inside electrodes from GITT characterization data, a method that is still widely used. However, in practice, the assumption for applying the method and its formula is not met for all battery electrode materials and their associated GITT characterization data. This aspect is observed in the case of $Na_{3}V_{2}{\left(PO_{4}\right)}_{2}F_{3}$ positive electrode materials.

A good alternative to determine the ion transport properties within electrode materials of LIBs and SIBs when the aforementioned assumption is not met is to resort to numerical simulations of GITT characterization data using either electrical equivalent circuit models or electrochemical pseudo-two-dimensional (P2D) models [32,33,34,35,36]. These modeling frameworks enable the quantification of parameter values as the diffusion coefficient in the solid phase of battery porous electrodes, *D_s_*, and the kinetic rate constant parameter, *k*, in the case of pseudo-two-dimensional electrochemical modeling. A reasonable agreement of the model predictions compared to the experimental data is shown in these previous works.

However, so far, the following were observed: (a) *D_s_* and *k* values could not be determined for all GITT steps, particularly for the ones located in the middle of the GITT characterization data; (b) the number of iterations needed to determine *D_s_* and *k* could be potentially reduced if a more efficient extraction process of these parameter values was developed; (c) the simulations associated with the determined *D_s_* and *k* do not always mimic the experimental data. These aspects can be attributed to the fact that the obtained parameter estimates are the ones that exhibit the lowest error between the experimental GITT step data and the simulations implementing all the pre-defined *D_s_* and *k* value combinations within a grid, which might not be a computationally efficient method or enable determining appropriate and physically relevant parameter values for all the GITT steps.

With this work, an innovative and efficient methodology is developed and presented in this paper. This methodology is based on a physics-informed algorithm that implements unsupervised machine learning to cluster all the GITT steps, whose outcomes serve as input to steer a P2D model for the simulations of each step and the extraction of their corresponding *D_s_* and *k* values. In the end, this method enables the extraction of both *D_s_* and *k* profiles for all the steps of the entire GITT characterization. Moreover, this method also demonstrates to be fast and hence efficient, considering that it does not require simulating the whole duration of each GITT step or many iterations, owing to the effort set in this work toward the efficiency optimization of the parameter extraction process.

## 2. Materials and Methods

### 2.1. Materials

#### 2.1.1. Electrochemical Characterizations

A $Na_{3}V_{2}{\left(PO_{4}\right)}_{2}F_{3}$ positive electrode material referred to as NVPF was considered for the GITT characterizations and the modeling works presented in this paper. The diameter of the active material particles is defined according to the particle size distribution (PSD), in the range between 1.2 µm and 14.7 µm.

A CR2032 coin cell or button cell casing, 20 mm in diameter and 3.2 mm in height, was used for the characterization in a half-coin cell (HCC) configuration of the electrode vs. sodium metal. The thickness of the positive electrode is equal to 90 µm, the thickness of the Na metal counter electrode sheet or negative electrode equals 100 µm, and the Sigma-Aldrich (Saint Louis, United States of America) Whatman separator has a thickness of 1 mm. The electrolyte is NaPF6 (1M) in EC: DMC (1:1).

GITT characterization was performed at 25 °C during the discharge or sodiation (insertion of Na^+^ in the positive electrode) process of the NVPF electrode. A total of 47 titration steps were performed. Each titration step was defined by a galvanostatic current pulse equivalent to C/25 or 122 μA for 30 min (i.e., low current value for a short time), followed by a relaxation period (i.e., no current applied) with a duration allowing it to reach a potential variation of less than 1 mV·h^−1^. In this way, a stable equilibrium potential was reached, which therefore enabled us to derive accurately the Open Circuit Potential curve of the electrode.

**Figure 1:**
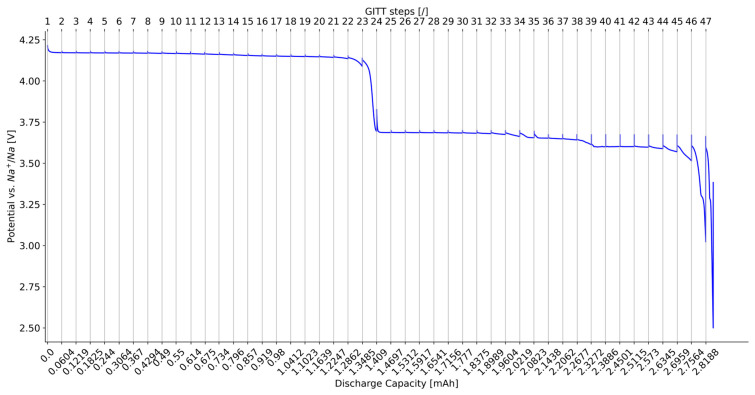
NVPF electrode potential vs. discharge capacity during GITT characterization at 25 °C.

The experiments were carried out with a BioLogic (Claix, France) electrochemical workstation while keeping the electrode in climate chamber conditions at 25 °C. The evolution of the electrode potential with respect to both discharge capacity and GITT steps is illustrated in Figure 1. The total discharge capacity of the GITT characterization is equal to 2.81 mAh.

In addition, three pre-GITT characterization cycles were performed at 25 °C, with a low C/20 C-rate following the Constant Current–Constant Voltage (CCCV) discharge mode. According to this protocol, both Constant Current (CC) charge and discharge are followed by a Constant Voltage (CV) step. The electrode potential is held at V_max_ and V_min_ at the end of both charge and discharge until the current flowing through the electrode is lower than the current corresponding to C/20 (with C the capacity at the characterization temperature 25 °C).

#### 2.1.2. Pseudo-Two-Dimensional (P2D) Electrochemical Modeling

In this work, GITT simulations were carried out with a pseudo-two-dimensional (P2D) electrochemical type of model. This model was developed and implemented in MATLAB by means of the Livelink for MATLAB interface of the finite element software COMSOL Multiphysics 5.2a. This model enables the simulation of the potential of the studied NVPF electrode for each of the 47 GITT titration steps.

**Figure 2:**
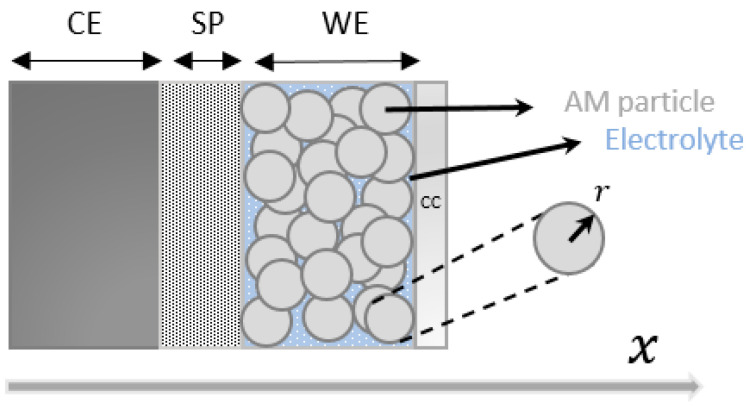
Schematic of the half-coin cell for P2D modeling.

The schematic of the half-coin cell considered for P2D modeling is illustrated in Figure 2. The working electrode (WE) refers to NVPF, the counter electrode (CE) refers to sodium metal, and SP and CC refer to the separator and aluminum current collector, respectively. Two dimensions in space are considered: the macroscopic x direction across the half-cell, and the microscopic radial direction across the electrode particles. The NVPF electrode is considered porous, associated with two solid and electrolyte phases. The solid phase is assumed to be made of uniformly distributed spherical particles of the same size. Hence, calculation of the effective conductivity and diffusivity parameters via Bruggerman’s correlation is allowed. Further, it is considered that no volume change occurs in the active material particles of the NVPF electrode during the insertion mechanism of Na^+^, and the voltage drop across the aluminum current collector is negligible. The temperature of the half-cell is considered to remain constant during the discharge or sodiation of the NVPF electrode.

Table 1 summarizes the governing nonlinear partial differential equations (PDEs) and their constitutive equations involved in the P2D electrochemical modeling [32,37,38,39]. Table A2 and Table A3 describe the corresponding subscripts and superscripts, and the Greek and Roman letters, respectively.

The Open Circuit Potential (OCP) of the NVPF electrode as a model parameter is derived from the last potential measurement of each GITT titration step. The electrolyte ionic conductivity, diffusion coefficient, and transference number are derived from [40].

#### 2.1.3. K-Means Unsupervised Machine Learning

The k-means algorithm, as part of unsupervised machine learning techniques, is a well-known and widely used clustering method. Clustering in data science allows the finding in a dataset of the greatest similarity within the same cluster and the greatest dissimilarity between different clusters [41]. In this work, a k-means unsupervised machine learning algorithm was implemented in Python via scikit-learn (version 1.2.2) with the sklearn.cluster module and the KMeans submodule for the clustering of unlabeled data. This aims at the categorization of the 47 GITT titration steps according to clusters based on their voltage characteristics.

The k-means algorithm clusters data by attempting to distinguish samples in *n* groups of equal variance, while minimizing a criterion known as the inertia or Within-Cluster Sum-of-Squares. The k-means algorithm divides a set *X* of *N* samples into *K* disjoint clusters *C*. The mean of each cluster, $\mu_{j}$, is commonly called the “centroid”. The centroids are defined in order to minimize the inertia, or the Within-Cluster Sum-of-Squares criterion (WCSS) in Equation (1):

(1) $$\sum_{i=0}^{n}\underset{\mu_{j}\in C}{\min}{\left(\left\|x_{i}-\mu_{j}\right\|\right)}^{2}$$

The clustering of data via the k-means machine learning algorithm involves three steps. The first step chooses the initial centroids based on *k* samples selected from the dataset *X*. Thereafter, follows two steps: a step to assign each sample to its nearest centroid and one to create new centroids based on the mean values of all the samples assigned to each previous centroid. These last two steps are repeated by the k-means algorithm until the difference between the old and the new centroids is less than a threshold and the centroids do not move significantly [42].

### 2.2. Methods

#### 2.2.1. Analytical Method

The galvanostatic intermittent titration technique consists of applying constant current pulses during a given time and measuring the potential response of the studied system.

Fundamentally, the chemical diffusion coefficient, D, in a system can be determined by GITT with Equation (2) (as long as $\tau \ll \frac{r^{2}}{D}$) [43,44] in the case where particles can be considered as semi-infinite solids:

(2) $$D=\frac{4}{\pi }{\left(\frac{1}{SFz_{A}}\right)}^{2}{\left(\frac{I_{0}\left(dE/dc\right)}{dE/d\sqrt{t}}\right)}^{2}$$

where *S*, *F*, and $z_{A}$ represent the particle area involved in the current pulses, the Faraday constant, and the charge number of the ion, respectively. $I_{0}$, *E*, and *c* designate the galvanostatic current step, the measured potential, and the surface concentration, respectively.

Basically, the determination of the diffusion coefficient with GITT characterization relies on several assumptions. For the Na^+^ diffusion coefficient in electrodes, one-dimensional semi-infinite particles are considered. Second, the transport of lithium is assumed to follow Fick’s law. Third, diffusion coefficients are assumed not to vary during the current pulses. For small and short current pulses, the dE/dc and $dE/d\sqrt{t}$ derivatives can be considered constant and approached by $∆E_{s}/∆c$ and $∆E_{\tau }/\surd t$.

Taking into account the relations between *S*, *F*, $z_{A}$*,* and $I_{0}$, the Na^+^ diffusion coefficient can be extracted from the data associated with each current pulse according to Equation (3):

(3) $$D_{s}={\left(\frac{\Delta E_{s}}{\Delta E_{\tau }}\right)}^{2}\frac{4L^{2}}{\pi \tau }$$

This equation was proposed by Weppner et al. and can be applied as long as the assumption for its use $\tau \ll \frac{L^{2}}{D_{s}}$ is satisfied [43,44]. In Equation (3), *L* represents the characteristic diffusion length equal to $R_{s}/3$ for spherical-like active material particles as the ones of NVPF [45,46], τ is the duration of the discharge current pulse, and $∆E_{s}$ and $∆E_{\tau }$ represent the changes in the steady-state potential and in the transient potential of the electrode.

**Figure 3:**
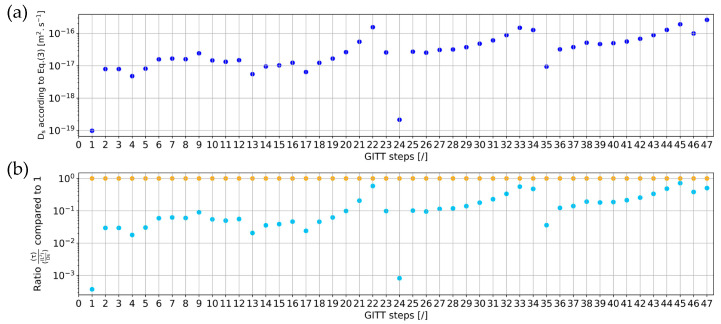
(**a**) Diffusion coefficient *D_s_* determined via Equation (3); (**b**) verification of the assumption associated with Equation (3).

Figure 3a shows the results of the diffusion coefficient D_s_ determined with Equation (3) for all the GITT steps of the NVPF positive electrode material. The values range from $10^{-19}$ to ${2\times 10}^{-16}$. It can be observed that for most of the GITT steps, τ is not significantly lower than $\frac{L^{2}}{D_{s}}$, with a ratio in Figure 3b ranging between 0.01 and 1, which was, however, the condition for the determination of D_s_ via Equation (3).

#### 2.2.2. Numerical Method

A numerical approach based on P2D electrochemical modeling for the determination of the diffusion coefficient *D_s_* and the reaction rate constant *k* for each GITT step via simulations is of high relevance. As mentioned in Section 1, a first approach would be to initially define a vector with possible values of *D_s_* and *k* for the whole set of GITT steps, then carry out simulations while looping over all these pre-defined *D_s_* and *k* value combinations. In the end for each step, the combination of *D_s_* and *k* leading to the lowest error between the experimental and simulation results is selected. In such an approach, the best *D_s_* and *k* values are purely determined based on the quantification of the simulation error when compared to the experimental data. As a consequence, the time it takes to test all the combinations might be considerably long, and for GITT steps associated with phase transitions in the electrode material, adequate *D_s_* and *k* values (allowing the reproduction of the experimental data) are not always included in the predefined vector of “guessed values”.

Therefore, it is important to consider, as in this work, that all the GITT steps and their corresponding *D_s_* and *k* values are interconnected. In this way, the determination of the *D_s_* and *k* values of step #n starts with the value extracted for step #n − 1. No specific upper or lower bounds, depending on whether we are lowering or increasing both parameters, have to be defined. In that way, it is therefore possible to extract adequate *D_s_* and *k* values for all of the steps of the GITT characterization data.

In practice, for each GITT titration step, first, a potential change occurs, referred to as the “current response” in this work. This is caused by the galvanostatic current pulse applied to the electrode initially in a near-equilibrium potential state. Thereafter, a second potential change is occurring, which is referred to as the “relaxation response” in this work. The latter results from the application to the electrode of a rest period, whose duration is longer than the “current response” to allow the electrode potential to return to an equilibrium state.

Fundamentally, the diffusion coefficient *D_s_* is linked to the relaxation response of a GITT step and further influences the current response and its slope. A low *D_s_* value leads the simulated current response to exhibit a high negative slope. A high *D_s_* value leads the current response to be more horizontal and flattened.

The reaction rate constant, *k*, only impacts the current response. In practice, an increase in the *k* value in the simulation shifts up the simulated current response. Similarly, a decrease in the *k* value shifts down the simulated current response. Changes in the *k* model parameter value purely result in a vertical upwards or downwards translation of the current response. The current response is sensitive to both *D_s_* and *k*, whereas the relaxation response is only sensitive to *D_s_*. Consequently, the approach for each GITT step in this paper consists of first proceeding to the extraction, via P2D simulations, of the diffusion coefficient in the solid phase *D_s_*, followed by the extraction of the reaction rate constant *k*.

**Figure 4:**
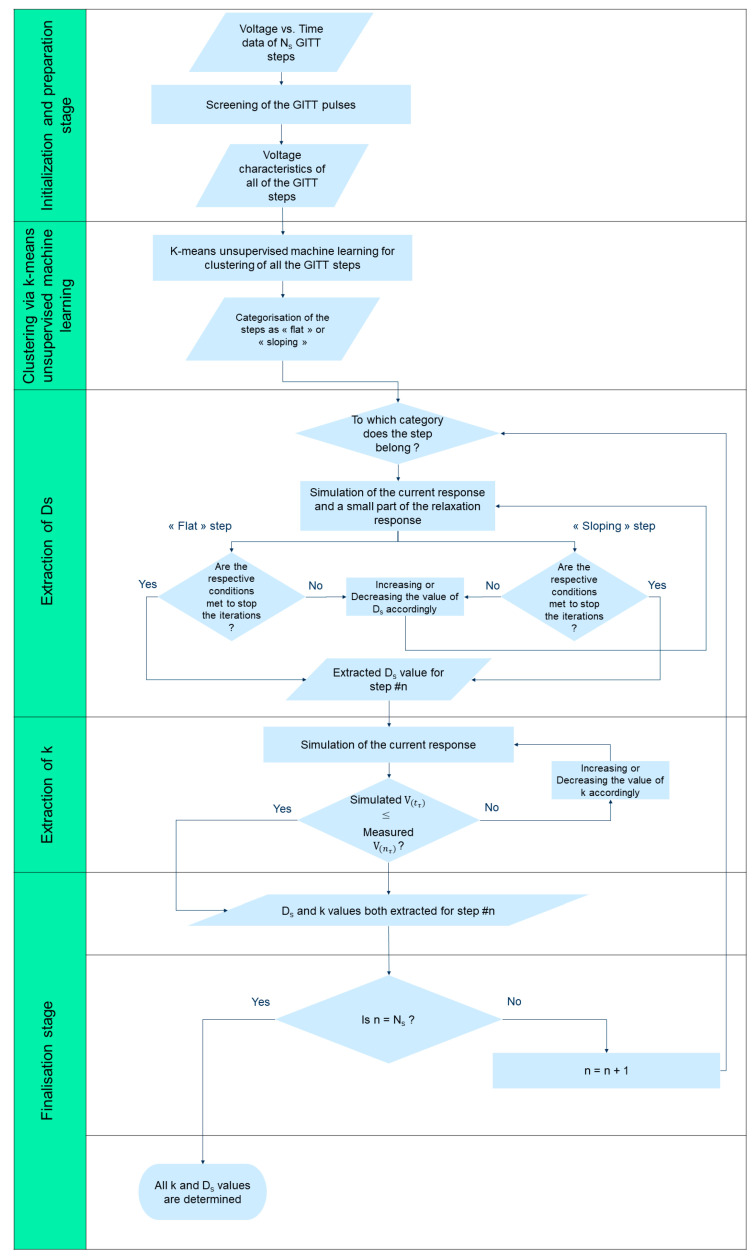
Flowchart of the developed methodology.

The flow diagram of the innovative and efficient methodology developed in this work is presented in Figure 4.

This methodology entails the following 5 stages: (1) initialization and preparation stage, (2) clustering via k-means unsupervised machine learning, (3) extraction of the diffusion coefficient *D_s_*, (4) extraction of the reaction rate constant *k*, (5) finalization stage. The initialization and preparation stage includes the screening of all the GITT steps and the extraction of their voltage characteristics for abstraction purposes for the next stage. In this stage, clustering via k-means unsupervised machine learning is performed based on the GITT step voltage characteristics. At this point, all the required information to conduct the extraction of both *D_s_* and *k* parameters via simulations is obtained. For these two extraction stages, a physics-informed algorithm is designed by implementing a P2D electrochemical model utilizing as input the results of the GITT step categorization of stage 2. In this way, the designed algorithm knows how to handle each of the steps. The finalization stage allows us to determine whether all the steps of the GITT characterization data have been processed.

K-means machine learning for clustering of the GITT steps is implemented as part of this methodology in stage 2, which enables us to distinguish between the “flat” steps on one hand and between the “sloping” or “non-flat” steps on the other. To conduct k-means machine learning clustering, each GITT step is abstracted and quantified by the difference between its first and its last measurement $V_{1N}=V_{\left(n=1\right)}-V_{\left(n=N\right)}$ and the difference between the second and last (i.e., at t=τ) measurement of its current response ${V_{1n_{\tau }}=V}_{\left(n=1\right)}-V_{\begin{array}{c} (n_{\tau })\; \end{array}}$. In practice, for each step, $V_{1N}$ relates to the relaxation response while $V_{1n_{\tau }}$ relates to the current response.

For sloping GITT steps, two conditions should be met to stop the iterations for the extraction of *D_s_*. First, all points of the current response exhibit a slope with the same order of magnitude. Second, the simulated voltage corresponding to the first measurement of the relaxation response is higher or lower than (whether we are iterating with positive or negative increments) or equal to the corresponding experimental value.

Two conditions should be met to stop the iterations for the extraction of *D_s_* in case of flat GITT steps. First, all points of the current response exhibit a slope with the same order of magnitude and the same sign. The iterations for the extraction of *k* are stopped in case the last simulated potential of the current response is lower than or equal to the corresponding measurement point. For each GITT step, the values of *D_s_* are incremented by 0.5% of the starting value at each new iteration, whereas the values of *k* are incremented by 0.2%.

For steps associated with a change in category from “flat” to “sloping” or “sloping” to “flat”, their *D_s_* and *k* values are expected to increase or decrease (whether we are iterating with positive or negative increments) more significantly compared to consecutive steps belonging to the same category. To speed up the process of *D_s_* and *k* extraction for these steps and reduce the number of iterations needed, increments up to 5% and 10% are defined, respectively.

## 3. Results

### 3.1. Potential vs. Time for All GITT Steps

**Figure 5:**
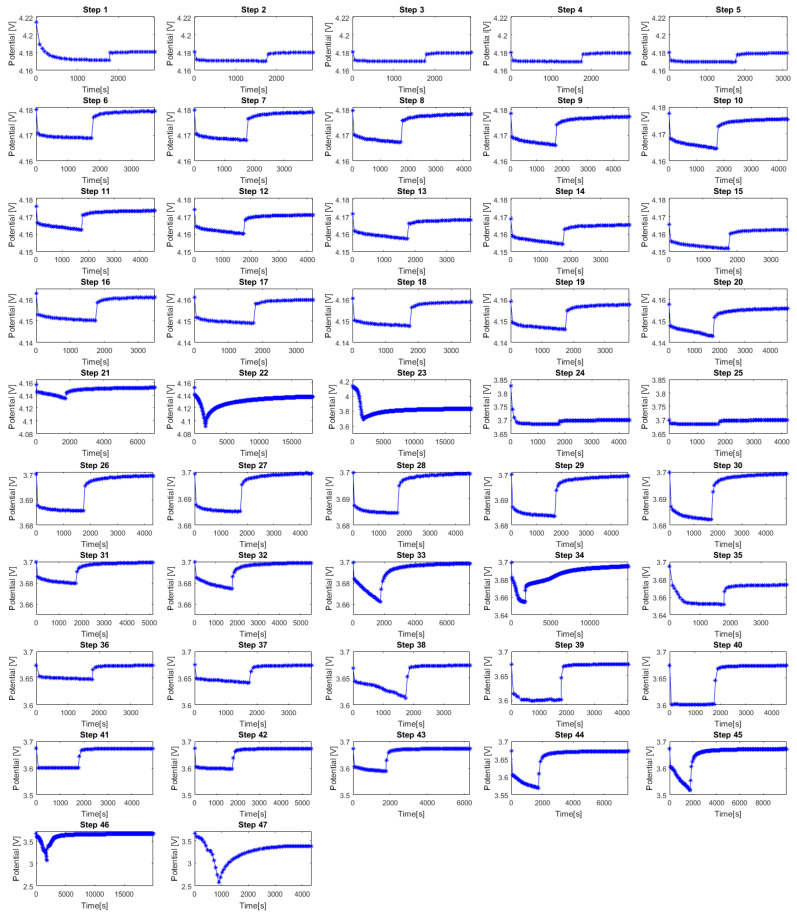
Overview of the GITT characterization from steps 1 to 47.

Figure 5 presents an overview of the evolution in time of the NVPF electrode potential for each GITT step from 1 to 47.

**Figure 6:**
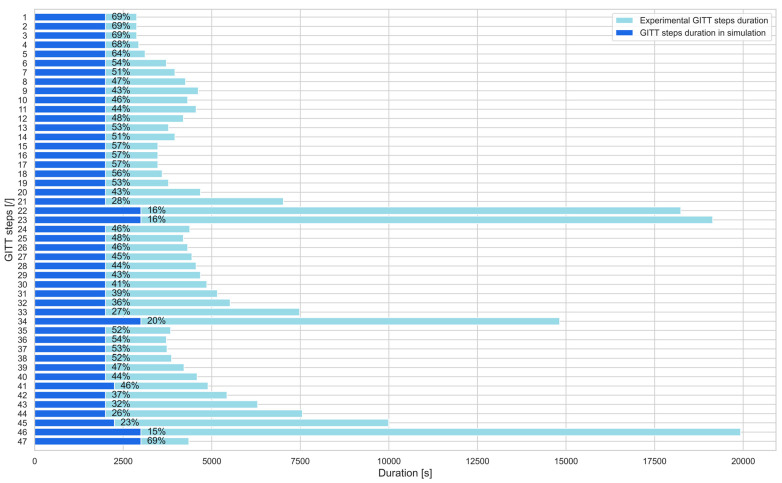
Duration of the GITT steps considered in the simulations and experimental characterization.

The decay in potential along with the GITT steps can be clearly observed in Figure 5. Several GITT steps such as 22, 23, 34, 46, and 47 exhibit nonlinear and steep current responses. This is linked to the occurrence of phase transitions occurring in the electrode material in the potential region of these steps. Consequently, the duration of the relaxation response needed for the potential to return to an equilibrium state is longer for these steps compared to the other GITT steps. This is illustrated with the light blue bars in Figure 6.

The dark blue bars in Figure 6 highlight the duration of the GITT steps used for simulation purposes. An advantage of this methodology is that the duration of the GITT steps to be taken into account in the simulations for the *D_s_* and *k* parameters’ extraction can be shortened compared to the experimental ones. On average, only 46% of the experimental step duration should be taken into account as an input parameter of the model to carry out the simulation of the GITT steps. For long steps associated with phase transitions occurring in the material, only 16% of the experimental step duration should be simulated. This enables a reduction in the time needed for simulating and extracting both *D_s_* and *k* parameters for each step, which reduces the total time required for the simulation of the entire GITT characterization.

### 3.2. Clustering of GITT Steps by K-Means Unsupervised Machine Learning

**Figure 7:**
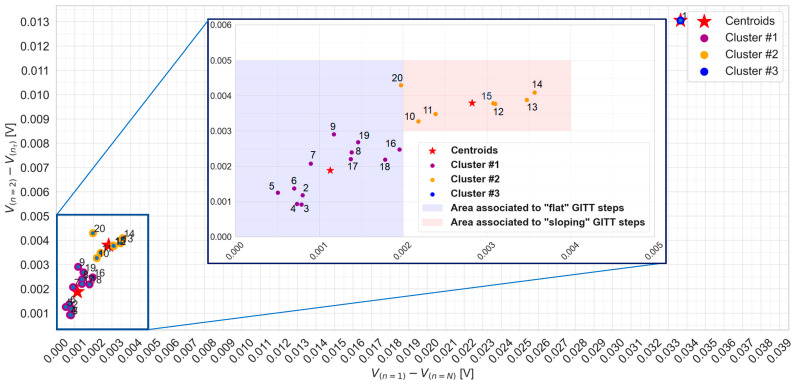
K-means clustering for GITT steps from 1 to 20.

Unsupervised k-means machine learning is achieved for the clustering of the 47 GITT steps according to their voltage characteristics abstracted via the differences $V_{1N}=V_{\left(n=1\right)}-V_{\left(n=N\right)}$ and ${V_{1n_{\tau }}=V}_{\left(n=1\right)}-V_{\begin{array}{c} (n_{\tau })\; \end{array}}$. Figure 7 presents an overview of the clustering of GITT steps 1 to 20. A total of three clusters and their centroids were identified. Particularly, step 1 is representing a cluster as such, with its x and y coordinates being those of the centroid itself. The two other clusters, clusters 1 and 2, comprise a total number of steps equal to 12 and 7, respectively.

In Figure 7, it can be observed that a difference $V_{1N}$ equal to 2 mV allows us to distinguish between cluster 1 and cluster 2. GITT steps with a difference $V_{1N}$ less than 2 mV and a difference $V_{1n_{\tau }}$ less than 3 mV are referred to as “flat” and belong to the purple area. Those with a difference $V_{1N}$ greater than 2 mV and a difference $V_{1n_{\tau }}$ greater than 3 mV are referred to as “sloping” or “non-flat” steps and belong to the red area. Steps belonging to cluster 2 are observed not to be all part of the same area, i.e., step 20 belongs to the purple area while all the others belong to the red area. According to these observations, GITT step 1 of cluster 3 can be classified as a sloping step. The area of flat GITT steps is defined by a set of 13 steps {{2–9},{16–20}}, while the area of sloping steps is defined by a set of 6 GITT steps {10–15}.

**Figure 8:**
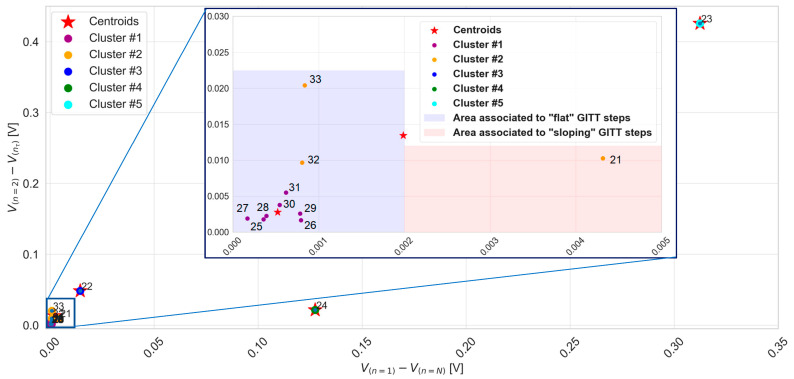
K-means clustering for GITT steps from 21 to 33.

Figure 8 presents an overview of the clustering of GITT steps 21 to 33. A total of five clusters and their centroids were identified. Particularly, steps 22, 23, and 24 are each representing one cluster as such, whose x and y coordinates are those of the centroids themselves. The two other clusters, clusters 1 and 2, comprise a total number of steps equal to 7 and 3, respectively. The centroid of cluster 2, associated with a difference $V_{1N}$ equal to 2 mV, delimits the flat and sloping GITT step areas. Steps belonging to cluster 2 are observed not to be all part of the same area, i.e., steps 32 and 33 belong to the purple area, while step 21 belongs to the red area. According to these observations, steps 22, 23, and 24 of clusters 3, 4, and 5 can be classified as sloping steps. The area of flat GITT steps is defined by a set of nine steps {25–33}, while the area of sloping steps is defined by a set of four GITT steps {21–24}.

**Figure 9:**
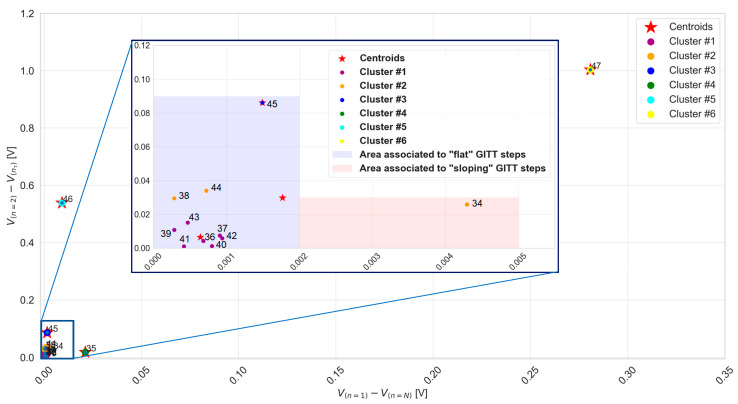
K-means clustering for GITT steps from 34 to 47.

Figure 9 presents an overview of the clustering of GITT steps 34 to 47. A total of six clusters and their centroids were identified. Particularly, steps 35, 45, 46, and 47 are each representing one cluster as such, whose x and y coordinates are those of the centroids themselves. The two other clusters, clusters 1 and 2, comprise a total number of steps equal to 7 and 3, respectively. The centroid of cluster 2, associated with a difference $V_{1N}$ equal to 1.8 mV, delimits the flat and sloping GITT step areas. Steps belonging to cluster 2 are observed not to be all part of the same area, i.e., steps 38 and 44 belong to the purple area, while step 34 belongs to the red area. According to these observations, steps 35, 45, 46, and 47 of clusters 3, 4, 5, and 6 can be classified as sloping steps. The area of flat GITT steps is defined by a set of 10 steps {36–45}, while the area of sloping steps is defined by GITT step {34}.

In this methodology, the clustering and further categorization of the GITT steps are achieved based on the implementation of k-means unsupervised machine learning. From the results in Figure 7, Figure 8 and Figure 9, a difference $V_{1N}$ less than or equal to 2 mV allows us to distinguish the areas of flat and sloping GITT steps.

Overall, the flat step area comprises a set of 32 steps {{2–9},{16–20},{25–33},{36–45}}, whereas the sloping step area comprises a set of 15 steps {{1},{10–15},{21–24},{34,35},{46,47}}.

**Figure 10:**
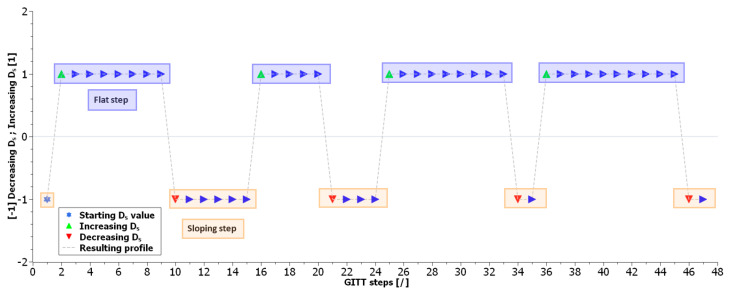
Categorization of the GITT steps by K-means unsupervised machine learning and resulting *D_s_* variations expected.

Figure 10 summarizes graphically these results with the use of the same purple and red colors as in Figure 7, Figure 8 and Figure 9. Throughout the entire GITT characterization, it can be observed that eight switches are occurring in total between the two categories of steps. These switches are corroborating the occurrence of phase transitions in the electrode material. In total, four category switches from sloping to flat steps occurring for steps {{1,2},{15,16},{24,25},{35,36}} and four category switches from flat to sloping steps occurring for steps {{9,10},{20,21},{33,34},{45,46}} are observed. In practice, sloping GITT steps exhibit a difference $V_{1n_{\tau }}$ higher than of the flat steps. Flat GITT steps exhibit a difference $V_{1N}$ lower than of the sloping steps. Therefore, a higher diffusion coefficient is associated with the flat steps compared to the sloping steps. Consequently, an increase in the diffusion coefficient *D_s_* value is expected while a switch from a sloping step to a flat step occurs. Conversely, a decrease in the diffusion coefficient *D_s_* is expected while a switch from a flat to a sloping step occurs. The resulting variations in the *D_s_* values are illustrated graphically in Figure 10.

## 4. Discussion

### 4.1. Diffusion Coefficient and Reaction Rate Constant Profiles

**Figure 11:**
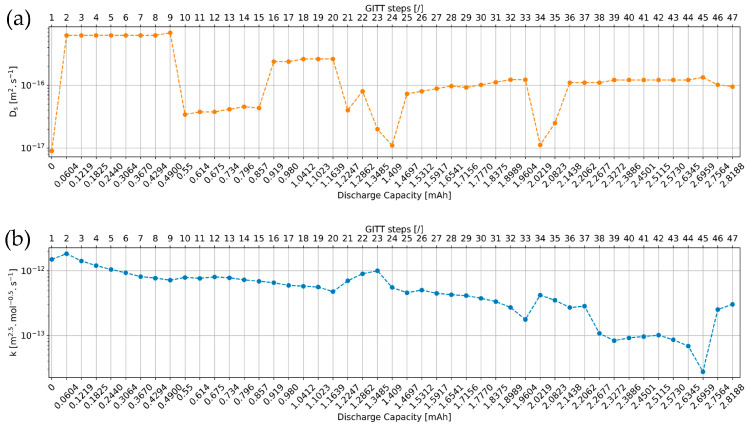
(**a**) Evolution of the diffusion coefficient Ds with the GITT steps and the discharge capacity; (**b**) evolution of the reaction rate constant k with the GITT steps and the discharge capacity.

The *D_s_* and *k* parameter values extracted from the simulations of the 47 GITT steps are illustrated in Figure 11. The Na^+^ diffusion coefficient and the reaction rate constant throughout the sodiation of the electrode are determined to vary within two orders of magnitude between $9\times 10^{-18}$ and $6.8\times 10^{-16}$ m^2^·s^−1^ and between $2.7\times 10^{-14}$ and $1.5\times 10^{-12}$ m^2.5^·mol^−0.5^·s^−1^, respectively. Despite differences in the material synthesis and composition available in the literature, it seems that analytical formulas intrinsic to GITT, EIS, and CV characterizations to derive Na^+^ diffusion coefficients in NVPF electrodes provide higher values in the ranges of [$10^{-14}$–$10^{-11}$] [47] (GITT), [$10^{-13}$–$10^{-12}$] [48] (EIS) and [$10^{-8}$–$10^{-7}$] [49] (CV), respectively, compared to those extracted via numerical simulations, as presented in this work.

In Figure 11a, when switching from a flat to a sloping step category (e.g., from step 9 to 10), as when switching from a sloping to a flat step category (e.g., from step 15 to 16), the value of *D_s_* clearly decreases and increases, respectively, in accordance with Figure 10. These switches are accompanied by changes in the order of magnitude of *D_s_*. These observations are reflecting the occurrence of phase transitions in the NVPF electrode material that can be identified using advanced XRD characterization techniques [50,51]. Several plateaus can be observed in the diffusion coefficient *D_s_* profile. These are generally lower in the second compared to the first part of the GITT characterization.

In Figure 11b, the profile of the reaction rate constant *k* exhibits an overall decreasing trend throughout the sodiation of the electrode. In the case of plateaus in the diffusion coefficient profile, the reaction rate constant decreases continuously. In case of an increase or decrease in *D_s_* when switching from one plateau to the other, the reaction rate constant tends mostly to decrease or increase.

The overall decreasing trend observed in the profiles of *k* and *D_s_* highlights that more and more sodium ions are inserted in the NVPF active material particles during the sodiation process of the electrode, which slows down the insertion of additional sodium ions in remaining particles.

### 4.2. Number of Iterations

**Figure 12:**
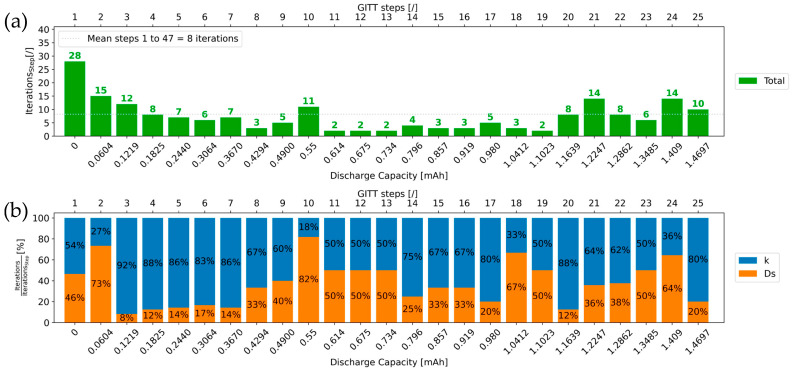
(**a**) Evolution of the number of iterations with the GITT step number and the discharge capacity; (**b**) normalized plot of the number of iterations for extracting *D_s_* and *k* values of steps 1–25.

**Figure 13:**
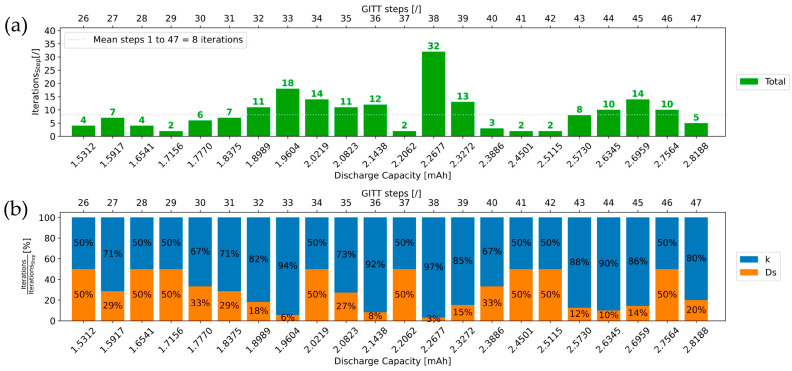
(**a**) Evolution of the number of iterations with the GITT step number and the discharge capacity; (**b**) normalized plot of the number of iterations for extracting *D_s_* and *k* values of steps 26–47.

Figure 12 and Figure 13 show the number of iterations needed to extract *k* and *D_s_* values for GITT steps 1 to 25 and 26 to 47, respectively. Overall, only eight iterations are required on average for the physics-informed algorithm to extract the *D_s_* and *k* values of a GITT step. Importantly, each of the iterations performed contributes to coming closer and closer to the final extracted *D_s_* and *k* values. With this methodology, each iteration is thus relevant and necessary. Since no “unnecessary” iterations are required, the parameter extraction process is time-efficient. In Figure 12 and Figure 13, it can be observed that 64% of all GITT steps exhibit less than eight required iterations, the average value. For most of the steps, the algorithm does not require more than eight iterations to extract both corresponding *k* and *D_s_* parameter values.

The number of iterations for the first GITT step in Figure 12 is relatively high. This is due to the fact that no prior step exists, which does not allow for starting the first iteration of the parameter extraction process with close enough values for *D_s_* and *k*. For this step, the starting value for *D_s_* is equal to $1\times 10^{-19}$ m^2^·s^−1^ from Figure 3a, and *k* is equal to $5\times 10^{-14}$ m^2.5^·mol^−0.5^·s^−1^. In Figure 13, GITT step 38 also exhibits a high number of iterations. This is mostly due to the number of iterations needed to extract its corresponding k value. In Figure 10, step 38 belongs to the flat GITT steps category like its previous step 37 and following step 39. This step is not associated with a switch in the step category such that no significant changes in the *k* value are assumed compared to that of the previous steps. Therefore, a small standard increment will be used for conducting the iterations. In view of the significant decrease in *k* occurring between steps 37 and 38, more iterations will be needed for this step.

Figure 12b and Figure 13b show that for 67% of all the GITT steps, the share of iterations (with respect to the total number of iterations) to extract the *k* value is higher than what is needed for *D_s_*. Thus, for most of the steps, the number of iterations needed to extract the final *k* value is higher than what is needed to determine the final *D_s_* value. This is in line with the continuous decreasing trend with local variations in the *k* profile in Figure 11b. In particular, more iterations for the extraction of *D_s_* and *k* final values would be initially required for GITT steps associated with a switch in the step category. In practice, around 10 iterations are needed for these steps. This results from the definition for these steps, of greater increments used for the iterations, achieved as part of the optimization of the methodology.

### 4.3. Comparison between Experimental and Simulation Data

**Figure 14:**
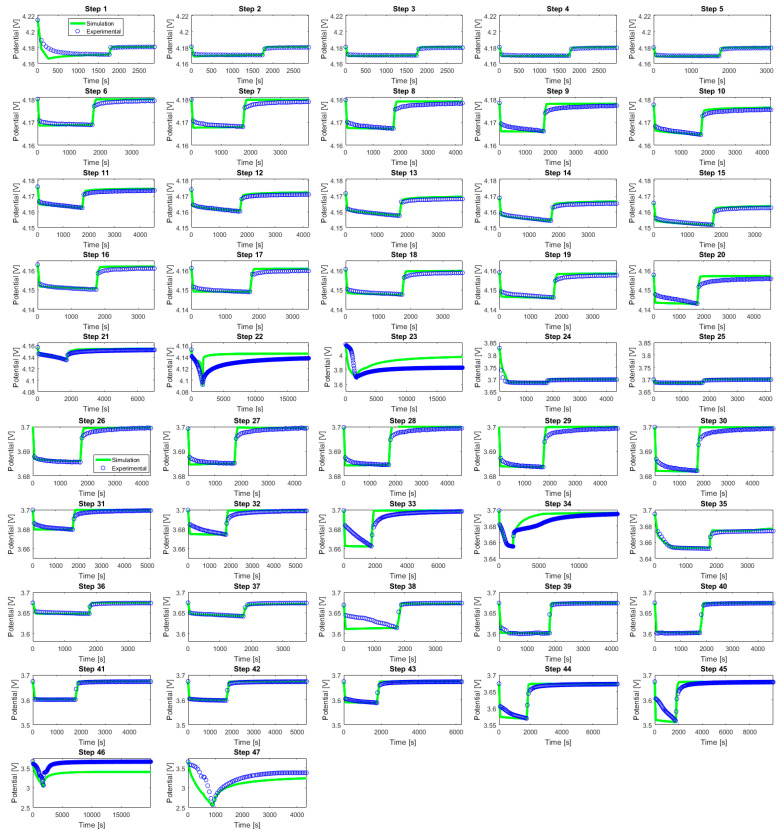
Comparison simulation and experimental data for GITT steps from 1 to 47.

The simulation results compared to the experimental data for GITT steps 1 to 47 are illustrated in Figure 14.

Overall, a good agreement was found between the simulated (green) and the experimental (blue) curves. This highlights the capability of the developed physics-informed algorithm to steer and allow the electrochemical P2D model to simulate and extract *D_s_* and *k* values for all 47 GITT titration steps.

### 4.4. Accuracy of the Simulation Compared to the Experimental Data

**Figure 15:**
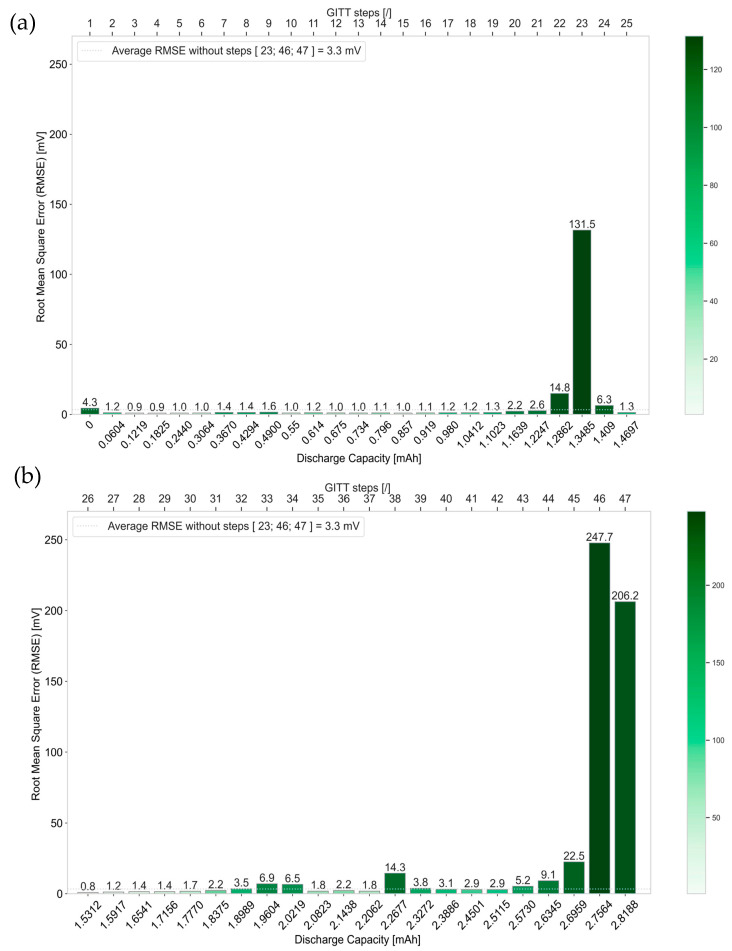
RMSE of simulation data for (**a**) GITT steps 1 to 25; (**b**) GITT steps 26 to 47.

Figure 15a,b illustrates the Root Mean Square Error (RMSE) values associated with steps 1 to 25 and 26 to 47, respectively.

The accuracy of the simulation results for each of the 47 GITT steps is quantified with the calculation of the RMSE. The latter quantifies the deviation of the results from the simulation of each GITT step, implementing the corresponding final extracted *D_s_* and *k* values, compared to the experimental data. Overall, an average value of 3.3 mV RMSE is determined, discarding outlier steps 23, 46, and 47. Around 70% of the GITT steps exhibit an RMSE below this average value. This means that for most of the GITT steps, the simulations implementing the respective final corresponding *D_s_* and *k* parameter values do not deviate more than 3.3 mV from the experimental data.

In Figure 15, the RMSE values associated with the steps in the middle, 23, and at the end, 46 and 47, of the GITT characterization are shown to be relatively high. This is due to the fact that causes of nonlinearities are not captured in the framework of the P2D modeling and its assumptions used in this work. The high RMSE values associated with the last GITT steps 46 and 47 are in line with the sudden and unexpected increase in the reaction rate constant *k* in Figure 11b for these steps.

### 4.5. Implementation of D_s_ and k Profiles in the Case of Constant Current Discharge Simulation

**Figure 16:**
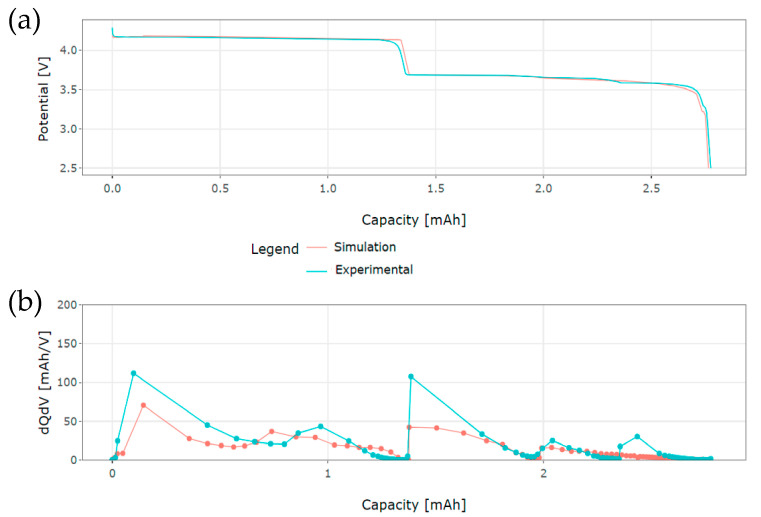
(**a**) Simulation and experimental results during C/20 constant current discharge; (**b**) Incremental Capacity (dQdV) profiles of simulation and experimental results.

The extracted *D_s_* and *k* profiles in Figure 16 are now implemented in the model to simulate a C/20 constant current discharge of the NVPF positive electrode at 25 °C. For implementation in the model, the reaction rate constant profile was fitted with a third-order polynomial. Due to the high RMSE values associated with the simulations of GITT steps 46 and 47 in Figure 15, their associated reaction rate constant values were not taken into consideration. Figure 16a shows the simulation results compared to the experimental data. An RMSE equal to 32 mV is calculated, which confirms the good agreement observed between the simulation and the experimental results.

Figure 16b illustrates the Incremental Capacity or dQdV curves associated with both simulation and experimental results. Overall, the peaks of both curves are well aligned with each other. A total of five peaks can be distinguished for the experimental data, whereas four peaks are defined for the simulation. In particular, no peak in the dQdV curve of the simulation is seen to correspond to the last one observed in the dQdV curve of the experimental data. This might originate from the rather flat behavior in this region of the Open Circuit Potential characteristic curve used as input in the model.

The existence of peaks in dQdV curves indicates phase transitions in electrode materials [52]. A correspondence can be made between the locations of the peaks of the dQdV curves in Figure 16b and the existing plateaus in the profile of *D_s_* in Figure 11a. This verifies the assumption that the occurrence of these plateaus in the profile of *D_s_* is related to phase transitions in the NVPF electrode material.

## 5. Conclusions

With this work, an innovative and efficient methodology is developed for the determination of the solid-state diffusion coefficient in electrode materials with phase transitions for which the assumption of applying the well-known formula from the work of Weppner et al. is not satisfied.

This methodology is defined by the implementation of a k-means machine learning screening of Galvanostatic Intermittent Titration Technique (GITT) steps, whose outcomes feed a physics-informed algorithm, the latter involving a pseudo-two-dimensional (P2D) electrochemical model for carrying out the numerical simulations.

As a result, *D_s_* and *k* parameter values were successfully extracted in this order for each of the 47 steps of the GITT characterization data during the discharge of an NVPF electrode. The Na^+^ diffusion coefficient and the reaction rate constant throughout the sodiation of the electrode were determined to vary between $9\times 10^{-18}$ and $6.8\times 10^{-16}$ m^2^·s^−1^ and between $2.7\times 10^{-14}$ and $1.5\times 10^{-12}$ m^2.5^·mol^−0.5^·s^−1^, respectively.

This innovative methodology presents for the first time the successful application of unsupervised machine learning via k-means clustering for the clustering of GITT steps according to their characteristics in terms of voltage. Based on this, all the steps could be further categorized as being “flat” or “sloping”.

This methodology also proved to be efficient. An average number of only eight iterations is sufficient to extract both *D_s_* and *k* associated with a GITT step. This achieves a significant reduction in the number of iterations required and hence speeds up the extraction process of the parameters, as compared to the existing literature. For most of the GITT steps, that is, 67% of them, the number of iterations needed to extract the reaction rate constant *k* value is higher than what is needed to extract the diffusion coefficient *D_s_* value.

In addition, as part of this methodology, the whole duration of each GITT step should not be simulated anymore and hence can simply be restricted to the current response and a small part of the relaxation response. For the longest steps, only 15% of their experimental duration has to be simulated.

When comparing the simulation of the GITT steps to their experimental data, an average Root Mean Square Error (RMSE) of 3.3 mV is determined. Most of the steps, that is, 70% of them, do not exhibit more than the average value of 3.3 mV deviation between the simulated and the experimental data.

The extracted *D_s_* and *k* profiles were further implemented for the simulation of C/20 constant current discharge of the NVPF electrode. An overall alignment of the peaks in both Incremental Capacity curves and a 32 mV RMSE demonstrate the good agreement observed between the simulation and the experimental results. A correspondence can be made between the locations of the peaks of the dQdV curves and the existence of plateaus in the profile of the diffusion coefficient *D_s_*. Hence, this verifies the assumption that the occurrence of these plateaus in the profile of *D_s_* is related to phase transitions in the NVPF electrode material.

Considering that this methodology and its physics-informed algorithm only rely on “if” and “else” statements, no particular module/toolbox is required. This enables its replication and implementation for electrochemical models written in any programming language and makes it generically applicable.

## Figures and Tables

**Table 1:** Physics and governing equations involved in the P2D electrochemical modeling [32,37,38,39].

Physics	Governing Equations	Mathematical Expressions
**Electrochemical reaction** **kinetics**	Butler–Volmer equation	$j={a_{s}i}_{0}\{exp\left(\frac{\alpha_{a}\eta F}{RT}\right)-exp\left(-\frac{\alpha_{c}\eta F}{RT}\right)\}$
	Electrode overpotential	$\eta =\left(\phi_{s}-\phi_{e}\right)-U$
	Exchange current density	$i_{0}=k\cdot {F\cdot \;\left(c_{e}\right)}^{\alpha_{a}}\cdot {\left(c_{s,max}-c_{s,surf}\right)}^{\alpha_{a}}\cdot {\left(c_{s,surf}\right)}^{\alpha_{c}}$
**Charge conservation**	Solid phase	$\nabla \cdot \left(\sigma^{eff}\cdot \nabla \phi_{s}\right)-j=0$
	Electrolyte phase	$\nabla \cdot \left(\kappa^{eff}\cdot \nabla \phi_{e}\right)+\nabla \left(\kappa_{D}^{eff}\nabla ln\left(c_{e}\right)\right)+j=0$
**Mass transfer**	Species conservation in solid phase	$\frac{\partial ({\epsilon_{s}c}_{s})}{\partial t}=\frac{D_{s}}{r^{2}}{\frac{\partial }{\partial r}(r}^{2}\frac{\partial c_{s}}{\partial r})$
	Species conservation in electrolyte	$\frac{\partial \left(\epsilon_{e}\cdot c_{e}\right)}{\partial t}=\nabla \cdot \left(D_{e}^{eff}\nabla c_{e}\right)+\frac{1-t^{+}}{F}\cdot j$

## Data Availability

The data are not publicly available.

## Appendix A

### Appendix A.1. Abbreviations

The abbreviations in Table A1 are used in this work.

**Table A1:** Abbreviations.

Abbreviation	Full Name
CC	Constant Current
CV	Constant Voltage
CV	Cyclic Voltammetry
dQdV	Incremental Capacity
EIS	Electrochemical Impedance Spectroscopy
GITT	Galvanostatic Intermittent Titration Technique
HCC	Half-coin cell
LIB	Lithium-ion battery
NVPF	$\mathrm{Sodium}\;\mathrm{vanadium}\;\mathrm{fluorophosphate},\;Na_{3}V_{2}{\left(PO_{4}\right)}_{2}F_{3}$
OCP	Open Circuit Potential
P2D	Pseudo-two-dimensional
PSD	Particle Size Distribution
RMSE	Root Mean Square Error
SIB	Sodium-ion battery
WCSS	Within-Cluster Sum-of-Squares
XRD	X-ray diffraction

### Appendix A.2. Subscripts and Superscripts

The subscripts and superscripts in Table A2 are used in this work.

**Table A2:** Subscripts and superscripts.

Symbol	Name
a	Anodic
A	Area
c	Cathodic
e	Electrolyte
eff	Effective
max	Maximum
S	Steady State
s	Solid
surf	Surface

### Appendix A.3. Greek and Roman Letters

The subscripts and superscripts in Table A3 are used in this work.

**Table A3:** Greek and Roman letters.

Symbol	Name	Units
α	Charge transfer coefficient	[-]
a_s_	Specific interfacial area	[m^−1^]
c	Concentration of ions (Na^+^ or Li^+^)	[mol·m^−3^]
D	Diffusion coefficient	[m^2^·s^−1^]
ε	Volume fraction	[-]
F	Faraday constant	[C·mol^−1^]
i_0_	Exchange current density	[A·m^−2^]
j	Transfer current per unit volume	[A·m^−3^]
κ	Ionic conductivity	[S·m^−1^]
k	Reaction rate constant	[m^2.5^·mol^−0.5^·s^−1^]
η	Overpotential	V
Φ	Electric potential	[V]
r	Radial coordinate along the radius of the electrode-active material particles	[m]
R	Ideal gas constant	[J·mol^−1^·K^−1^]
σ	Electrical conductivity	[S·m^−1^]
τ	Pulse duration	[s]
t^+^	Transference number of ions (Na^+^ or Li^+^)	[-]
T	Temperature	[K]
U	Open Circuit Potential	[V]
